# Correspondence—"Cohesion of Molecules, and Their Resistance to Displacement Constituting Hardness"

**Published:** 1881-05

**Authors:** 


### ARTICLE VII.

### *Correspondence.*

Mr. Editor.—Under the head of "Correspondence" in the March number of your journal, I notice some very interesting questions by J. W. F., and as I don't exactly understand the drift of your answer to him, I would like to call your attention to them again, and also ask some other questions suggested by that answer. J. W. F., asks if the "cohesion of molecules and their resistance to displacement" is not what constitutes hardness in any thing. Now is not that really the proper and a satisfactory explanation of hardness? And is not the softness produced by annealing due to the fact that the heat has driven the particles farther apart and thus given room for their condensation when force is applied? You say, "If we would for convenience sake call annealed gold adjustable foil and the variety known as cohesive foil unadjustable, these would satisfy us just as well as any other terms." Either there is a typographical error here or you found yourself getting tired at this point of your editorial duties, for cohesive foil and annealed foil are usually supposed to be the same. But to which variety did you mean to apply the term *un*adjustable? The advocates of the two kinds will, with equal truth, claim that each foil is perfectly adjustable when properly manipulated. In fact, if either were really unadjustable to a cavity, it would be valueless as a material for permanent fillings.

Can you tell me why anyone should speak of *welding* cohesive gold—as you say, "all pure gold is cohesive," but none of it is weldable. It seems to me that this is an error in our "yet new, and as a consequence, not exact nomenclature" which is liable to lead to serious mistakes; for example, I once heard a professor state to a class of dental students that cohesive foil could not be *welded properly* except by a blow. As I had seen experienced dentists work cohesive foil satisfactorily by hand pressure, I could not see why he should say this, until he gave us as a reason that no one had ever seen a blacksmith try to weld two pieces of iron together by pressure. We might ask who ever saw a blacksmith attempt to weld together two pieces of iron 100,000th of an inch in thickness, as our gold leaf probably is; or who ever saw a blacksmith attempt to weld together two pieces of iron as cold as our gold is when we work it. Since all pure gold is cohesive, is not perfect contact the only thing necessary to its cohesion, and cannot this perfect contact be brought about by

*sufficient* hand pressure, properly applied, as well as by any mallet ever invented?

Very respectfully,

C. L. S.

---

In regard to the above questions of Dr. S., we reply that hardness and cohesion are not correlatives, as he will see if he reflects and compares things. The diamond is the hardest of substances, but flies asunder at a slight blow; lead is soft, yet tenacious. The specific gravity of platinum does not seem to be increased by annealing, which apparently should be the case were its "molecules driven further apart." It may be said that the density of the diamond is the cause of its hardness, but we find bismuth and antimony brittle as well, and a few grains of the latter will render many hundreds of grains of gold unmalleable and brittle; yet no one would say that the molecules of the latter were by the slight alloying made denser in arrangement. It is quite doubtful we think if gold foil under ordinary manipulation is indeed made denser by malleting, and no one we believe asserts that unannealed foil, fresh from the gold-beater has greater specific gravity than that which is annealed. We must remember that while molecules are convenient they are mythical, and we have really only a clever hypothesis to work on in their use. "Unannealed" foil was intended to be written in the paragraph to which attention is directed, though we do not feel always like blaming the printer, as the copy is not always clear, and written hurriedly. Of course the unannealed or non-cohesive foil was referred to as adjustable, comparatively speaking.

The "proper manipulation" referred to is so large a factor in the perfection of the filling as to overshadow all the others. Read the disputes going on all the time about this.

As to "why any one should speak of *welding* cohesive foil," we search in vain in the article referred to for any statement that all pure gold is cohesive but none of it is weldable. We did state that some pure metals, as for instance platinum, are only weldable at a white heat, as is iron. On the other hand, clean surfaces of gold, lead, etc., will cohere perfectly cold. Gold is eminently *the* weldable metal. But it is an error for any one, even "a professor" to "state to a class that cohesive foil could not be welded properly except by a blow." He might have had the idea in his mind that it (gold) could be *best* welded by a blow, just as the smith can best weld his iron by blows. But iron is often welded by pressure, as in puddling mills, gun-barrel shops, etc. Where *perfect welding* is required, in a small way, pressure will perhaps be found inefficient; besides which the conditions are not just equal, as the "smith must strike while the iron is hot."

You are correct in asserting that perfect contact is all that is needed to insure cohesion: the method is a matter of dispute. Still if you will stick a tack in the floor of your office, take a half ounce hammer and try to *push* it or *press* it up to the head, and then *drive* another along side of it with blows of the same hammer, it may illustrate the wonderful force of "impact" as contrasted with simple "pressure." The question is not so much what the hand-pressure *may be* capable of in comparison with malleting, as what usually results. Clamp two smooth pieces of steel together in a vise—an old pair of bullet moulds are excellent for the purpose—and drill a hole of any desired size so that half the cavity is in either jaw; fill (*with cohesive foil, remember,*) first with hand pressure and then with the mallet; and report the result as to specific gravity, perfection of welding, and the mallet we feel sure will win. Remember we don't refer to non-cohesive gold in this proposed experiment.—EDITORS.

